# Differential Outcomes and Biologic Markers of Radiation-Associated vs. Sporadic Osteosarcoma: A Single-Institution Experience

**DOI:** 10.3389/fonc.2019.01523

**Published:** 2020-01-22

**Authors:** Brittany L. Siontis, Jonathan B. McHugh, Emily Roberts, Lily Zhao, Dafydd G. Thomas, Dawn Owen, Laurence H. Baker, J. Sybil Biermann, Scott M. Schuetze, Rashmi Chugh

**Affiliations:** ^1^Department of Internal Medicine, University of Michigan, Ann Arbor, MI, United States; ^2^Department of Pathology, University of Michigan, Ann Arbor, MI, United States; ^3^Biostatistics Department, School of Public Health, University of Michigan, Ann Arbor, MI, United States; ^4^Department of Radiation Oncology, University of Michigan, Ann Arbor, MI, United States; ^5^Department of Orthopedic Surgery, University of Michigan, Ann Arbor, MI, United States

**Keywords:** radiation-induced neoplasms, sporadic osteosarcoma, radiation-associated osteosarcoma, secondary malignancy, metastatic tumor antigen-1 (MTA-1), ezrin

## Abstract

**Background:** Radiation-associated osteosarcoma (RAO) is a rare, life-threatening complication from radiation. Many physicians presume RAO has a worse prognosis than sporadic osteosarcoma (SO), although limited objective data exist. We conducted a retrospective study comparing these entities.

**Methods:** We identified adults treated at our institution with osteosarcoma (1990–2016) and categorized tumors as SO or RAO based on location within a prior radiation field. We extracted data on demographics, treatment and primary malignancy and examined available tumor samples for MTA-1 and ezrin using immunohistochemistry (IHC).

**Results:** Of 159 identified patients, 28 had RAO, diagnosed at a median interval from radiation of 11.5 years (1.5–28 years). Median follow-up was 2.8 years (0.1–19.6 years). Median progression free survival (PFS) and overall survival (OS) were not significantly different in the small population of patients with metastases, SO (*n* = 20) vs. RAO (*n* = 6): PFS 10.3 months vs. 4.8 months (*p* = 0.45) and OS 15.6 months vs. 6.1 months (*p* = 0.96), respectively. For the larger group with localized disease, median relapse-free survival (RFS) and OS were significantly different, NR vs. 12.2 months (*p* < 0.001) and NR vs. 27.6 months (*p* = 0.001) in SO (*n* = 111) vs. RAO (*n* = 22), respectively. On IHC, there were significant differences in distribution of high, intermediate or low MTA-1 (*p* = 0.015) and ezrin (*p* = 0.002) between RAO and SO tumors.

**Conclusions:** Patients with metastases at diagnosis fared poorly irrespective of prior radiation. RAO patients with localized disease had worse outcomes without detectable differences in therapy rendered or treatment effect in resected specimens. Higher expression of MTA-1 in RAO patients may suggest an underlying difference in tumor biology to explain differences in outcomes.

## Introduction

Osteosarcoma is a rare, primary bone tumor that has a bimodal distribution of incidence with a peak in children and adolescents and a second, smaller peak in the elderly. The relationship between radiation therapy and subsequent development of osteosarcoma was first recognized in 1922 in patients who received external beam radiation therapy for tuberculous arthritis (1). As the use of radiation therapy for the management of cancer has increased, so have reports of radiation-associated osteosarcoma (RAO). The latency period between radiation therapy and diagnosis of RAO is highly variable, often 10–15 years after radiation therapy (2, 3).

Historically, RAO has been thought to carry a poor prognosis based on retrospective analyses, with a reported disease free survival as low as 17% (2). Known risk factors contributing to a worse prognosis include metastatic disease at diagnosis, incomplete or no resection and tumor size >5 cm (4). In addition, it is postulated that prior chemotherapies, elderly status of functional status, may limit optimal treatment for patients with RAO. While there have been several series describing treatment and outcomes of RAO, there is little objective data directly comparing demographics, treatment and outcomes of RAO to sporadic osteosarcoma (SO). McHugh et al. reported on the difference between primary vs. radiation-associated craniofacial osteosarcoma (5). Craniofacial osteosarcoma carries an overall favorable prognosis compared to its appendicular counterpart. In this series, 47% of *de-novo* craniofacial OS were high-grade with 80% of patients alive without disease. Alternatively, all radiation-associated tumors were high grade, all patients experienced recurrent disease and half of the patients died of their disease.

Several factors associated with more aggressive tumor biology have been described in osteosarcoma. Ezrin, a cytoskeleton linker protein involved in regulation of growth, has been associated with metastatic potential and poor prognosis in mouse models of osteosarcoma (6).

Metastatic tumor antigen-1 (MTA-1) promotes migration, invasion and survival of human keratinocytes (7). Elevated levels of MTA-1 in breast cancer enhances metastasis, increases motility and potentiates growth (8). In a series of 53 osteosarcoma specimens, MTA-1 was expressed in 81% of high-grade tumor samples, but in none of the low grade tumors (9). P53 participates in regulation of cell cycle and apoptosis and plays a role in cancer pathogenesis (10). Multiple series evaluating p53 expression in craniofacial osteosarcomas noted increased expression in high-grade tumors (11–13). Furthermore, there is some suggestion that *TP53* gene mutations, which are often accompanied by p53 overexpression, play a role in post-radiation osteosarcoma (14). Ki67 serves as a maker of cell proliferation and is used as a prognostic factor in multiple cancer types. McHugh et al. noted higher Ki67, p53, and ezrin expression in radiation-associated craniofacial osteosarcoma compared to sporadic tumors (5).

We conducted a retrospective study comparing demographics, therapy and outcomes of SO to RAO at our institution with the aim of better understanding the differences in natural history and treatments rendered. We conducted immunohistochemistry (IHC) studies to evaluate differences in markers of aggressiveness to identify differences in biology and behavior of these entities.

## Materials and Methods

### Patient Identification

The University of Michigan Electronic Medical Record Search Engine (EMERSE) (15) was searched using the term “osteosarcoma” to identify patients with a diagnosis of osteosarcoma treated at our institution between 1990 and 2016. Patients under age 18 were excluded given concern for potential differences in biology of adult vs. pediatric sporadic osteosarcoma. Furthermore, given the latency between radiation and development of osteosarcoma, there were unlikely to be pediatric patients in the RAO cohort. Patient medical records were reviewed, and tumors were characterized as sporadic or radiation-associated based on a history of prior radiation within the field of osteosarcoma. Details regarding demographics, clinical presentation, pathologic features, treatment protocols, outcomes, and primary malignancy in the setting of radiation-associated tumors were extracted from clinical records. All research was approved by the University of Michigan Institutional Review Board (HUM00068553).

### Pathology

Available representative tumor samples were obtained and reviewed by a sarcoma pathologist to confirm the diagnosis and assess tumor grade. Immunohistochemical staining for Ki67, MTA-1, p53, and ezrin were conducted. Immunohistochemical staining was performed on the DAKO Autostainer (DAKO, Carpinteria, CA) using Envision+ or liquid streptavidin-biotin and diaminobenzadine (DAB) as the chromogen. De-paraffinized sections were labeled with the antibodies for 30 min at ambient temperature. Microwave 10 mM citrate, pH6 epitope retrieval was used prior to staining for both antibodies. Appropriate negative (no primary antibody) and positive controls were stained in parallel with each set of slides studied. Ki67 was reported as percentage of tumor nuclei positive. All other results are given as a modified Allred intensity score (0, 1 = weak; 2 = moderate, and 3 = strong).

### Statistical Analysis

Descriptive statistics, such as median and range, were calculated for continuous variables; frequencies were presented for categorical variables. To compare two categorical variables, a frequency table was created and analyzed using the Chi-square test of independence or Fisher's exact test. Continuous variables were compared using *t*-tests. Tumor size was log-transformed for statistical testing. Progression-free survival (PFS) and overall survival (OS) from date of diagnosis was estimated by the Kaplan-Meier method and compared using the Log-rank test. Cox proportional hazards regression models were created for RAO association, adjusting for patient age at diagnosis, metastatic status, tumor size on the logarithmic scale, and an interaction effect for metastatic status and RAO association. Statistical significance was defined as a two-sided *P* < 0.05. All analyses were conducted using SAS (version 9.4, SAS Institute, Cary, NC).

## Results

We identified 159 patients with osteosarcoma, of which 28 had RAO (Table 1). Median follow-up for all patients was 2.8 years (range 0.1–19.6 years). Median follow-up for survivors was 5.3 years (range 1–19.6). RAO patients were older (*p* < 0.001) and had smaller tumors (*p* = 0.003) at diagnosis than SO patients. The most common location for metastatic disease at diagnosis or at progression was bone and lung. There were no differences in the location of metastases between the two groups. The most common primary cancers for which patients received radiation therapy were breast cancer, prostate cancer and lymphoma, as well as retinoblastoma in two patients. The median radiation dose was 60 Gy and a median latency period of 11.5 years.

**Table 1:** Patient demographics and tumor characteristics.

**Variable**	**SO**	**RAO**	***p*-value**
Age, median (range)	38 (18–79)	61 (18–77)	**<0.001**
Male, n (%)	73 (56)	16 (57)	0.891
Tumor size (cm), median (range)	8.7 (1.8–20)	5.6 (2–12)	**0.003**
Location of metastases at diagnosis or progression/relapse	*n* = 60	*n* = 20	
Bone	29 (48)	12 (60)	0.37
Lung	45 (75)	12 (60)	0.20
Time from radiation to osteosarcoma, median years (range)		11.5 (1.5–28)	
Radiation dose, Median Gy (range)		60 (44–75.8)	

*Bolded values highlight p-values which were found to be significant (<0.05)*.

### Localized Disease

Twenty-two RAO and 111 SO patients had localized disease at diagnosis (Table 2). RAO tumors were more prevalent in the axial skeleton while SO tumors were more prevalent in the appendicular skeleton (*p* < 0.001). Nineteen (86.4%) RAO patients and 104 (93.7%) SO patients underwent resection (*p* = 0.24). RAO and SO patients received a similar number of chemotherapy cycles, with a median of 5 and 6 cycles, respectively (*p* = 0.55). While patients in both groups received doxorubicin-based regimens, SO patients received more cisplatin and methotrexate (*p* = 0.03) while RAO patients received more carboplatin (*p* < 0.001). There was no difference in patients achieving <10% viable tumor from pre-operative chemotherapy in resected specimens (*p* = 0.75).

**Table 2:** Localized disease at diagnosis.

**Variable**	**SO**	**RAO**	***p*-value**
	**(*n* = 111)**	**(*n* = 22)**	
Primary Location, *n* (%)			
Axial	34 (31)	18 (82)	**<0.001**
Spine	10 (9)	5 (22)	
Head and neck	16 (14)	8 (36)	
Chest wall	8 (7)	5 (23)	
Appendicular (upper/lower extremity)	77 (69)	4 (18)	**<0.001**
1st line chemotherapy, *n (*%)			
Neoadjuvant + Adjuvant	72 (65)	14 (64)	0.91
Adjuvant alone	20 (18)	4 (18)	0.99
# chemotherapy cycles, median (range)	6 (2–7)	5 (3–7)	0.55
Chemotherapy Regimen, *n* (%)			
Cisplatin/doxorubicin (CD)	18 (16)	8 (36)	**0.03**
Methotrexate/cisplatin/doxorubicin (MAP)	28 (25)	1 (5)	**0.03**
Doxorubicin/Ifosfamide (AI)	12 (11)	2 (9)	0.81
CD alt AI	23 (21)	1 (5)	0.07
Carboplatin/doxorubicin	1 (1)	5 (23)	**<0.001**
Resection, *n* (%)	104 (94)	19 (86)	0.24
<10% viable tumor, *n* (%)	16/54 (30)	3/12 (25)	0.75
Location of relapse	*n* = 43 (39)	*n* = 14 (64)	
Local	13 (30)	5 (36)	0.70
Distant	23 (54)	5 (36)	0.25
Both	7 (16)	4 (29)	0.32

*Bolded values highlight p-values which were found to be significant (<0.05)*.

**Figure 1:**
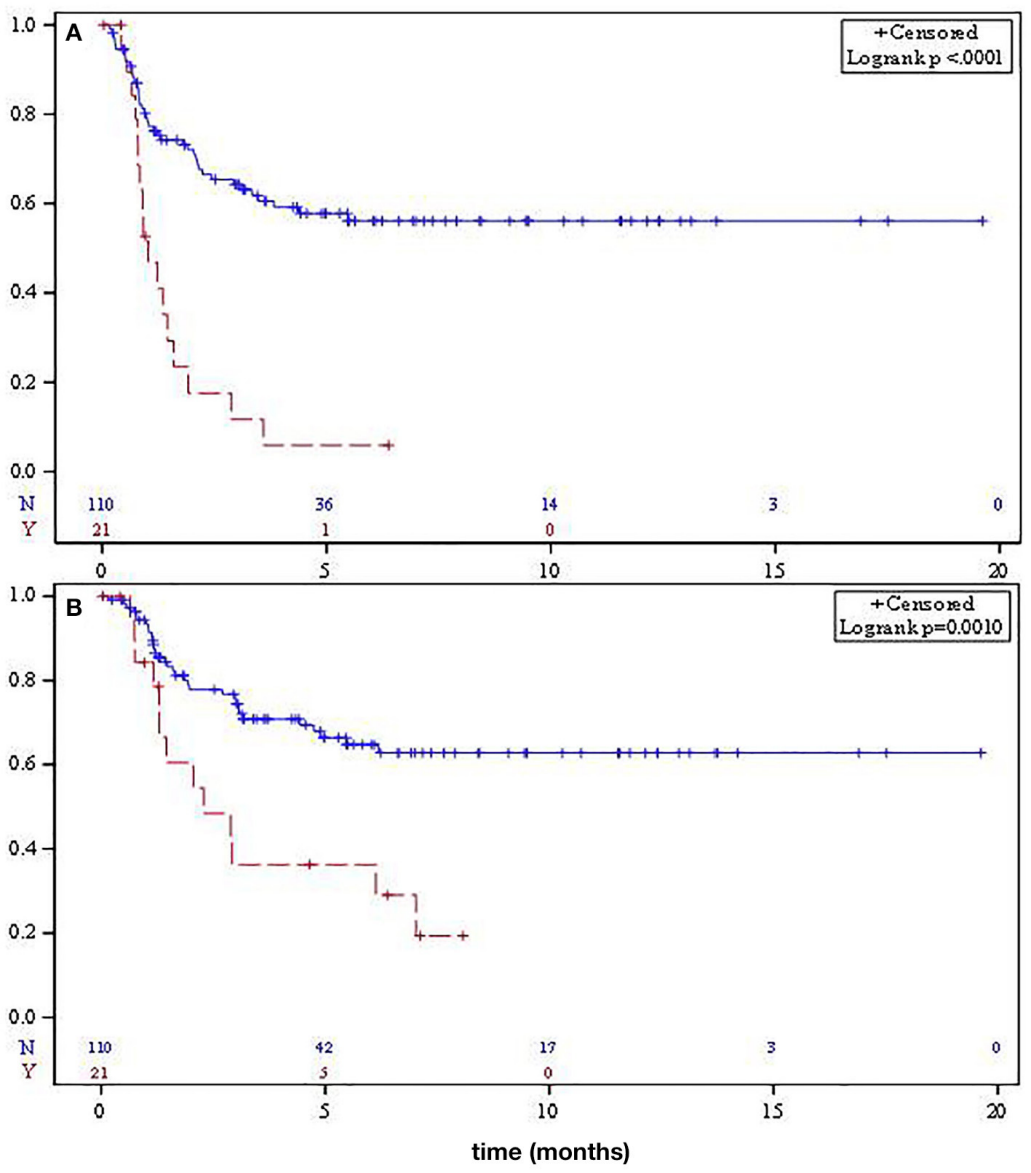
Median relapse-free survival **(A)** and overall survival **(B)** for localized sporadic osteosarcoma (solid) and radiation-associated osteosarcoma (dashed).

Median relapse-free survival (RFS) was significantly different for localized SO and RAO patients at not reached and 12.2 months, respectively (*p* < 0.001, Figure 1A). Median OS for SO patients was not reached, while median OS for RAO patients was 27.6 months (*p* = 0.001, Figure 1B). Adjusting for age and tumor size, RAO portended a worse PFS (HR 2.92, 95% CI 1.51–5.69) and OS (HR 2.46, 95% CI 1.20–5.07).

### Metastatic Disease

Twenty patients with SO and 6 patients with RAO had metastatic disease at diagnosis. Resection of the primary tumor was performed in 11 (55%) SO patients and 2 (33.3%) RAO patients (*p* = 0.35). Metastasectomy was completed in 7 (35%) SO patients and 2 (33.3%) RAO patients (*p* = 0.94). Patients with SO received a median of 2 lines of chemotherapy (range 0–6) compared to 1.5 lines (range 0–4) in RAO patients (*p* = 0.46).

**Figure 2:**
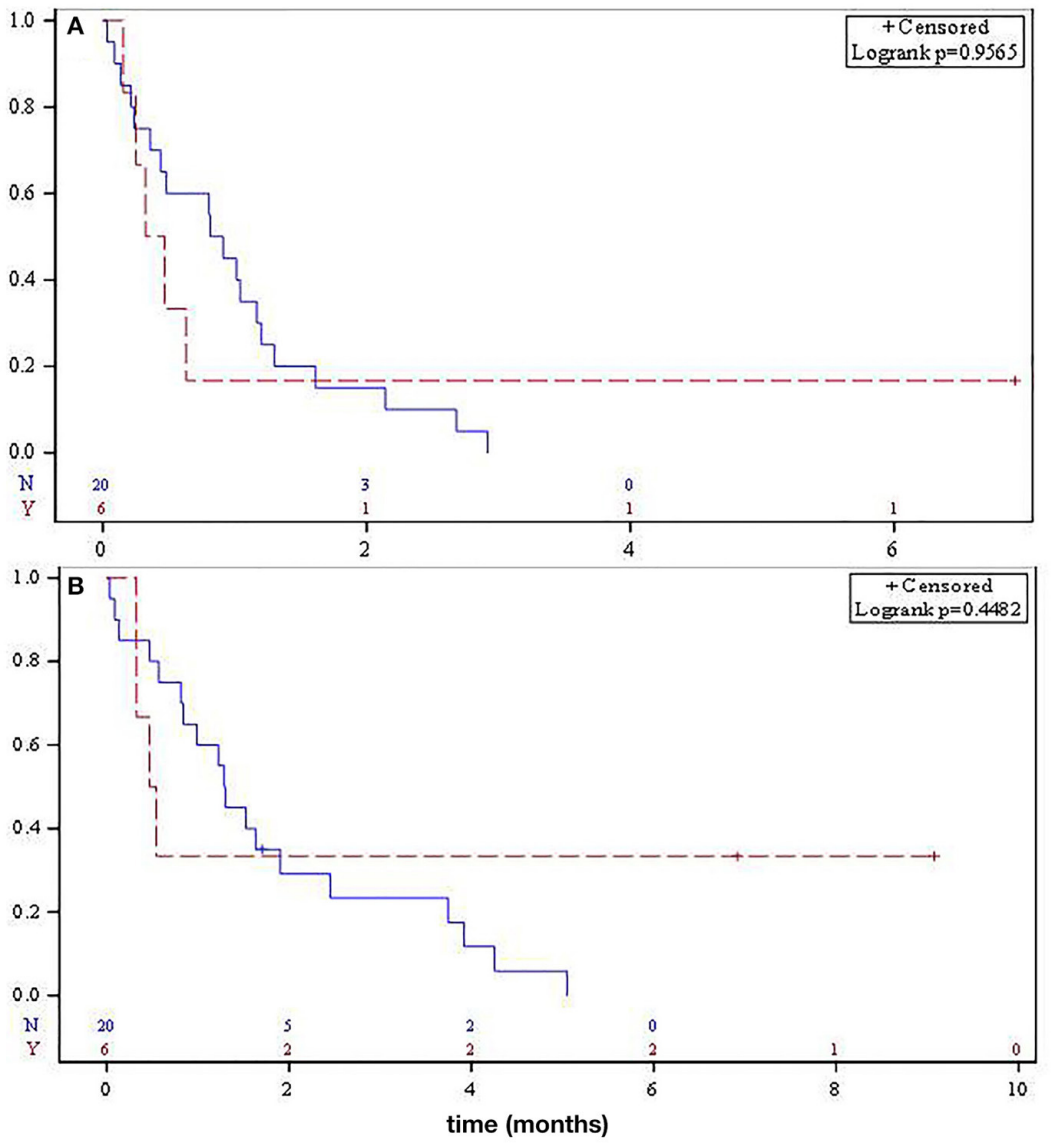
Median progression-free survival **(A)** and overall survival **(B)** for metastatic sporadic osteosarcoma (solid) and radiation-associated osteosarcoma (dashed).

There was no statistical difference in median PFS for SO and RAO disease, 10.3 and 4.8 months, respectively (*p* = 0.95, Figure 2A). Median OS was not significantly different between the two groups (15.6 vs. 6.1 months, respectively, *p* = 0.45, Figure 2B).

### Pathology

**Figure 3:**
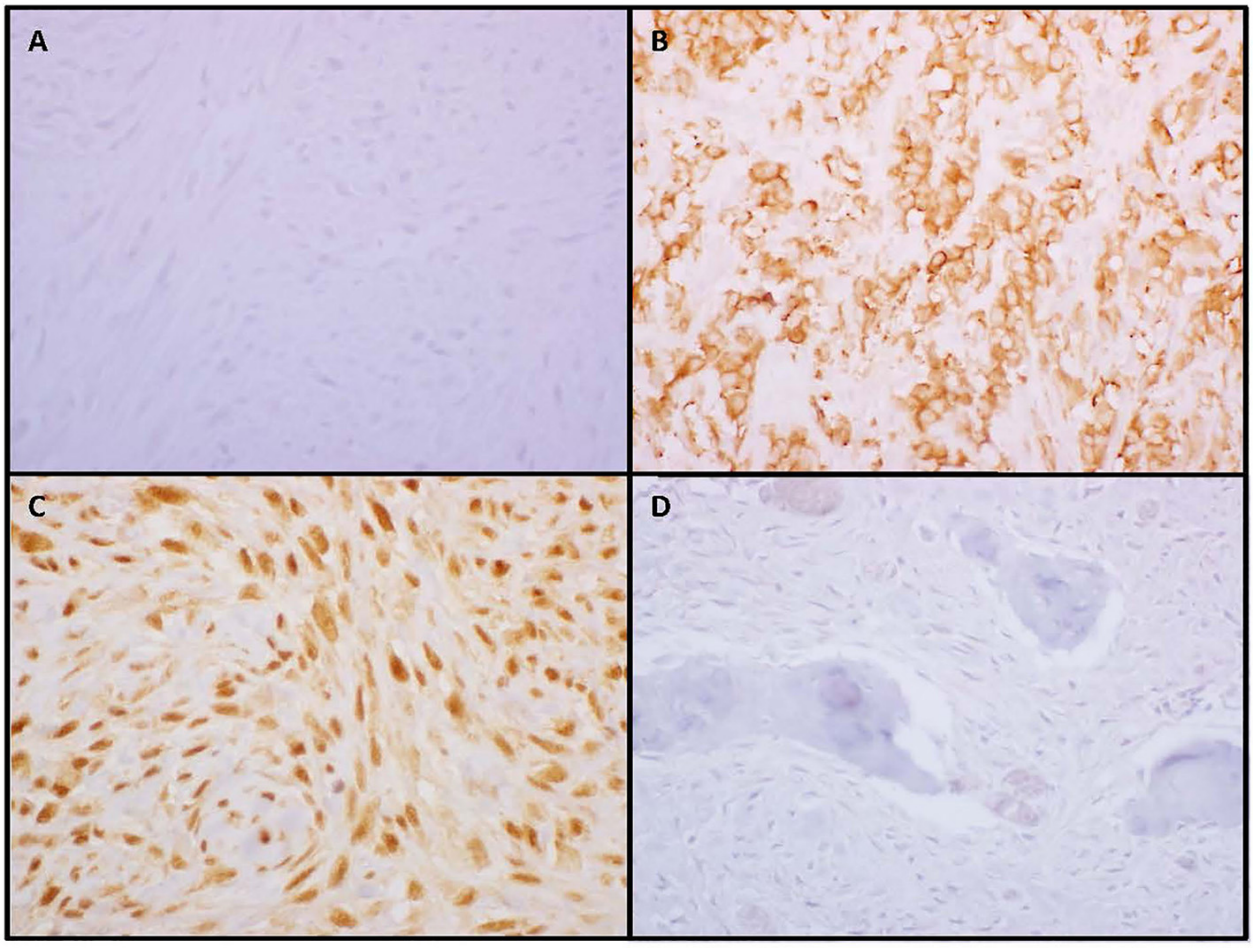
Low or negative ezrin expression **(A)** was more common in sporadic osteosarcoma while high membranous expression **(B)** was more common in radiation-associated tumors. All radiation-associated osteosarcomas exhibited high nuclear metastatic tumor antigen-1 staining **(C)** while a subset of sporadic tumors were negative **(D)**.

Fifty SO and 14 RAO tumor samples were available for pathology review. Pathology characteristics are shown in Table 3. There were no differences in the grade, histologic subtype of osteosarcoma (osteoblastic, chondroblastic, or fibroblastic) or p53 IHC between the two groups. Interestingly, significant differences between SO and RAO were detected in tumor expression of MTA-1 (*p* = 0.015, Figure 3) and ezrin (0.002) with all RAO samples having high MTA-1 and most SO samples having low ezrin expression.

**Table 3:** Pathology.

**Variable**	**SO (*n* = 50)**	**RAO (*n* = 14)**	***p-*value**
Grade, n (%)			0.098
High	42 (84)	14 (100)	
Low	8 (16)	0 (0)	
Histologic subtype, n (%)			0.629
Osteoblastic	38 (76)	10 (71)	
Fibroblastic	3 (6)	2 (14)	
Chondroblastic	6 (12)	2 (14)	
Mixed	3 (6)	0 (0)	
Ki-67, mean (95% CI)	39.1 (29.7-48.6)	49.3 (32.5-66.1)	0.29
MTA-1, n (%)	*n* = 50	*n* = 12^*^	**0.015**
High	29 (58)	12 (100)	
Intermediate	8 (16)	0 (0)	
Low	13 (26)	0 (0)	
P53, n (%)	*n* = 44	*n* = 14	0.081
High	8 (18)	5 (36)	
Intermediate	1 (2)	2 (14)	
Low	35 (80)	7 (50)	
Ezrin, n (%)	*n* = 48	*n* = 14	**0.002**
High	8 (16)	5 (36)	
Intermediate	3 (6)	4 (28)	
Low	37 (77)	5 (36)	

* *Two samples omitted due to absence of tumor in analyzed sample. Bolded values highlight p-values which were found to be significant (<0.05)*.

## Discussion

Osteosarcoma is a rare but significant complication of radiation therapy that has historically been associated with a worse prognosis compared to its sporadic counterpart. It is important to understand the natural history and prognosis of this rare entity to appropriately manage our patients and choose therapies. In our retrospective analysis, patients with metastatic osteosarcoma at diagnosis, irrespective of prior radiation therapy, had poor median progression-free and overall survival rates. Worse outcomes for metastatic osteosarcoma compared to localized tumors has been reported in the literature, including the EURAMOS trial in which 3-year event-free survival for patients with metastatic disease was significantly worse (16). These data highlight an unmet medical need to develop more effective drug therapy in this patient population. While prior radiation did not impact outcomes in metastatic disease, the sample size was relatively small with only six patients with metastatic RAO, making conclusions about metastatic population inconclusive. Patients with localized SO in our series had significantly improved RFS and OS rates compared to RAO.

RAO patients were significantly older than SO patients. This is not surprising given the median latency period between radiation therapy and the development of osteosarcoma being 10 years or greater in multiple studies including ours (2, 3). Older patients are more likely to have comorbidities that may impact chemotherapy choice. While doxorubicin administration was similar, RAO patients received more carboplatin and less cisplatin and methotrexate. Despite these differences, similar numbers of chemotherapy cycles were administered and examination of available tumors noted no difference in treatment effect as indicated by the number of patients with <10% viable tumor.

Interestingly, patients with RAO had smaller primary tumors than SO patients. RAO patients also have a history of prior cancer, and may undergo more routine imaging and physical examination, may be more apt to notice changes in a tumor bed and report symptoms earlier than younger patients with little past medical history.

RAO tumors were more commonly located in the axial skeleton while SO tumors were more commonly located in the appendicular skeleton. The difference in location of RAO vs. SO tumors is expected given the primary diagnoses for which patients received radiation therapy (breast cancer, prostate cancer, and lymphoma). In patients with localized disease who relapsed, there was no difference in the location of relapse (distant vs. local) between the two groups.

The obvious difference in outcomes of localized disease despite overall similarities in therapies rendered suggests an underlying difference in the biology of these tumors. Multiple markers of tumor aggressiveness have been identified in exploratory studies, and while not prospectively validated may provide insight into the biology of these tumors. In osteosarcoma mouse models, ezrin was associated with increased metastatic potential and poorer prognosis while high ezrin expression correlated with chemotherapy resistance (6, 17). Over-expression of MTA-1 has been noted in high-grade osteosarcoma tumor samples with little to no expression in low-grade samples (9). Mardanpour et al. evaluated tumor samples from 56 osteosarcoma patients and noted a correlation between increased p53 and Ki67 with worse PFS and OS (18). In 47 resected osteosarcomas following neoadjuvant chemotherapy, increased p53 expression correlated with worse overall survival while expression in biopsy samples was not predictive of chemotherapy response or survival (19). On IHC evaluation of p53, Ki67, and ezrin in RAO vs. SO craniofacial osteosarcoma, there was higher expression of all three in the radiation-associated tumors (5).

In our series, the majority of patients had high-grade tumors and there was no difference in p53 expression. However, the distribution of high, intermediate and low expression of MTA-1 and ezrin was significantly different between the two groups. A higher proportion of RAO patients had high MTA-1 while a higher proportion of SO patients had low ezrin expression. These data, while limited by the small number of tumor samples available for review, suggest there may be an underlying difference in the aggressiveness of RAO tumors, which may account for the significant difference in outcomes in localized disease despite similar treatment.

Metastatic osteosarcoma portends an overall poor prognosis irrespective of sporadic or radiation-associated disease, with the limited sample size in this study limiting conclusions in outcomes. Patients with localized SO have significantly better PFS and OS compared to patients with RAO despite similar therapies. Differences in outcomes and expression of biomarkers (such as MTA-1 and ezrin) suggest an underlying difference in the biology of radiation-associated tumors, as well as the necessity for alternative therapeutic strategies.

## Data Availability Statement

The datasets for this article are not publicly available. Requests to access the datasets should be directed to the corresponding author, Rashmi Chugh (rashmim@med.umich.edu).

## Ethics Statement

The studies involving human participants were reviewed and approved by University of Michigan Institutional Review Board. Written informed consent for participation was not required for this study in accordance with the national legislation and the institutional requirements.

## Author Contributions

BS and RC involved with the study concept and design, data acquisition, analysis, and interpretation as well as manuscript preparation. JM conducted the pathology review as well as immunohistochemistry testing and interpretation. DT assisted with IHC testing and interpretation. ER and LZ performed statistical analysis. DO, LB, JB, and SS assisted with manuscript editing and review.

### Conflict of Interest

The authors declare that the research was conducted in the absence of any commercial or financial relationships that could be construed as a potential conflict of interest. The reviewer BVT declared a past co-authorship with several of the authors LB, SS, and RC to the handling Editor.
